# Polymer-derived SiOC as support material for Ni-based catalysts: CO_2_ methanation performance and effect of support modification with La_2_O_3_

**DOI:** 10.3389/fchem.2023.1163503

**Published:** 2023-03-22

**Authors:** E. Szoldatits, J. Essmeister, L. Schachtner, T. Konegger, K. Föttinger

**Affiliations:** ^1^ Institute of Materials Chemistry, TUWien, Vienna, Austria; ^2^ Institute of Chemical Technologies and Analytics, TUWien, Vienna, Austria

**Keywords:** polymer-derived ceramics, methanation, carbon capture and utilization, silicon oxycarbide, catalyst stability

## Abstract

In this study, we investigated Ni supported on polymer-derived ceramics as a new class of catalyst materials. Catalysts have to withstand harsh reaction conditions requiring the use of a support with outstanding thermal and mechanical stability. Polymer-derived ceramics meet these requirements and bring the additional opportunity to realize complex porous structures. Ni-SiOC and La-modified Ni-SiOC catalysts were prepared by wet impregnation methods with target concentrations of 5 wt% for both metal and oxide content. Polymer-derived SiOC supports were produced using a photoactive methyl-silsesquioxane as preceramic polymer. Catalysts were characterized by N_2_-adsorption-desorption, XRD, SEM, H_2_-TPR, and *in-situ* DRIFTS. CO_2_ methanation was performed as a test reaction to evaluate the catalytic performance of these new materials at atmospheric pressure in the temperature range between 200°C and 400°C. XDR, H_2_-TPR, and *in-situ* DRIFTS results indicate both improved dispersion and stability of Ni sites and increased adsorption capacities for CO_2_ in La-modified samples. Also, modified catalysts exhibited excellent performance in the CO_2_ methanation with CO_2_ conversions up to 88% and methane selectivity >99% at 300°C reaction temperature. Furthermore, the pyrolysis temperature of the support material affected the catalytic properties, the surface area, the stability of active sites, and the hydrophobicity of the surface. Overall, the materials show promising properties for catalytic applications.

## 1 Introduction

Two of the biggest challenges we and upcoming generations have to face are global climate change due to anthropogenic CO_2_ production and the increasing energy consumption due to economic development and population growth. In order to reduce and avoid CO_2_ emissions caused by industrial processes and combustion of fossil fuels, the main strategy is to increase the share of renewable energy sources worldwide. Another promising strategy is the conversion of CO_2_ into valuable chemicals that function as fuels or raw materials. Here, recent efforts focus on the development of catalysts which exhibit high activity, selectivity and long-term stability for electrochemical, photocatalytic or thermal catalytic reduction of CO_2_ (Porosoff and Yan B, 2016; Sun et al., 2017; Jangam et al., 2020).

Methane is one of the most important energy carriers and chemical feedstocks. Currently delivered from natural gas and shale gas, the aim of industrial nations, especially in Europe, is to become independent from natural sources and to produce CH_4_ as synthetic natural gas (SNG) through the hydrogenation of CO_2_ released from industrial flue gases. The CO_2_ methanation reaction according to Eq. 1 was first described in 1902 by Paul Sabatier and Jean-Baptist Senderens (Frontera et al., 2017). Nowadays, this reaction experiences a renaissance as the production of CH_4_ from CO_2_ meets the increasing energy consumption, and is seen as a chance to mitigate industrial CO_2_ emissions by recycling them. Another advantage of methane as an energy carrier is the already existing infrastructure from natural gas pipelines (Frontera et al., 2017; Jangam et al., 2020).

$$CO_{2}+4\;H_{2}\to CH_{4}+{2\;H}_{2}O;∆H_{298K}=-164\;kJ/mol$$ (1)

Though the CO_2_ methanation reaction is exothermic and thermodynamically favourable at ambient pressure and at relatively low temperatures between 25°C and 400°C, there is a kinetic barrier, which is the reason why highly active and selective catalysts are needed. Owing to its ready availability, low cost and satisfactory catalytic performance, nickel (Ni) has been extensively investigated as a catalyst for the Sabatier reaction (Le et al., 2017; Shen et al., 2020; Yusuf et al., 2021). Some of the main drawbacks encountered in the use of Ni-based catalysts are the insufficient low-temperature activity, poor dispersion and reducibility of the catalyst particles, sintering and aggregation of the active phase. Several studies showed that La_2_O_3_ can be an effective additive to avoid particle coalescence and sintering (Zhi et al., 2011; Zhu et al., 2011; Mihet et al., 2021; Riani et al., 2021). Another simple and cost-effective solution to increase the stability of catalysts is the use of a support material with high thermal conductivity and thermal stability such as silicon carbide to dissipate the reaction heat (Zhi et al., 2011; Zhang et al., 2013). One possibility to produce these materials is the polymer-derived ceramics (hereafter PDC) route, where a silicon-based polymer is converted into a ceramic during a thermal treatment process (Colombo et al., 2010; Lale et al., 2018). This approach offers multiple advantages and opens up new possibilities, including:

• Versatile chemical compositions due to a broad variety of preceramic polymers (Colombo et al., 2010)
• Tailorable material properties such as introduction of hierarchical porosity (Colombo, 2008; Lale et al., 2018; Konegger et al., 2021), functionalisation of the ceramic with metal centres which can act as catalysts (Schubert et al., 2017; Fu et al., 2018; Macedo et al., 2019; Kaur et al., 2020; Schumacher et al., 2020) or introduction of particulate fillers like SiC to ameliorate mechanical and thermal stability (Essmeister et al., 2022)
• Photocurable PDC systems offering a high freedom in shaping as they are suitable for stereolithography-based additive manufacturing (Zanchetta et al., 2016; Fu et al., 2018; Schmidt and Colombo, 2018; Essmeister et al., 2022)

One of the versatile possible applications of PDCs is their use as catalyst carrier structures in heterogeneous catalysis (Konegger et al., 2015). Until now, there exist several studies about the preparation of metal-doped PDCs, some of them reporting on the preparation of Ni-containing PDCs with a focus on the application as catalysts for CO_2_ hydrogenation (Schubert et al., 2017; Macedo et al., 2019; Kaur et al., 2020; Schumacher et al., 2020). There also have been studies on the performance of Pt catalysts supported on polymer-derived Si-Al-C-N in NaBH_4_ hydrolysis reaction to form hydrogen (Majoulet et al., 2013; Lale et al., 2018). However, to the best of our knowledge, there exist no studies investigating the catalytic properties of impregnated Ni catalysts supported on SiOC derived from photocurable preceramic polymers.

## 2 Experimental section

### 2.1 Preparation of the preceramic polymer

The photocurable preceramic polymer system used in this work is based on a materials system recently established in our team for the development of additively manufactures SiOC/SiC composites (Essmeister et al., 2022). To prepare the preceramic polymer, 40 g methyl-silsesquioxane (Silres-MK, Wacker Industries) were dissolved in 20 g TPM (Tripropyleneglycol-methylether, Sigma Aldrich) at 70°C under constant stirring. When MK was fully dissolved, 10 g TMSPM [3-(trimethoxysilyl)-propyl methacrylate, Sigma Aldrich] were added as a crosslinker and the solution was stirred for 1 h at room temperature. To start the hydrolysis, a mixture of 0.2 g HCl (32 wt%) and 0.8 g TPM was added dropwise. After another 14 h under constant stirring and stripping of side products under 80 mbar at 40°C, 2 wt% of Genorad*16 (RAHN) were added to stabilize the polymer for storage. For further processing, 5 wt% MAA (Methacrylic acid, Sigma Aldrich) and 10 wt% TRIM (Trimethylolpropane trimethacrylate, Sigma Aldrich) were added to the resin as reactive diluents. The mixture was homogenized using a Thinky ARE-250 planetary mixer at 2000 rpm for 4 min and degassed at 800 rpm for 10 min. Then, 1 wt% BAPO [Phenyl-bis(2,4,6-trimethylbenzoyl)-phosphinoxide, RAHN] was added as a photoinitiator and the resin was homogenized again as described above. The photosensitive resin was cast into silicone moulds and exposed to light (405 nm) for 5 min.

To obtain the support material, the preceramic polymer platelets were converted into SiOC using controlled thermal treatment in argon atmosphere at 600 or 800°C, respectively. The used temperature profile was composed of initial burnout steps at 200°C (2 h) as well as 500°C (2 h) and further, 2 h at the maximum temperature. The heating rate was 0.5°C/min up to 200°C, then 1°C/min up to T_max_. The detailed temperature profile of pyrolysis can be seen in Supplementary Figure S1.

The pyrolyzed samples were grinded using a vibrating mill (Retsch MM 40) with ZrO_2_ inlet at a frequency of 30 s^−1^ until the grain size was <180 µm.

### 2.2 Wet impregnation

Catalysts containing 5 wt% Ni and La_2_O_3_, respectively, were prepared by wet impregnation using nickel nitrate hexahydrate (Ni(NO_3_)_2_*6H_2_O) and lanthanum nitrate hexahydrate [La(NO_3_)_3_*6H_2_O] as precursors. For a final Ni content of 5 wt%, an appropriate amount of Ni precursor (0.25 g per g support) was dissolved in deionized water, the support was added, and the solution was stirred at room temperature for 12 h. Then, the suspension was heated to 80°C until the water was fully evaporated, and the remaining powder was dried at 100°C for 12 h. The dried samples were calcined at 440°C for 4 h in air (SiOC800) or in argon atmosphere (SiOC600). Lanthanum-modified catalysts were prepared using the same procedure: After impregnation with lanthanum nitrate (0.1 g per g support) and calcination (4 h at 440°C in air or Ar atmosphere), the support was impregnated and calcined again with Ni as described above. The obtained samples are denoted as Ni(La)/SiOC_T, where T is the pyrolysis temperature of the support material, e.g., Ni/SiOC600 or NiLa/SiOC600.

### 2.3 Catalyst characterization

To determine specific surface area and porosity, N_2_ adsorption and desorption at −196°C was performed following standard BET (Brunauer-Emmet-Teller, five points between 0.03 and 0.5 p/p^0^) and BJH (Barrett–Joyner–Halenda) procedures using a Micromeritics ASAP2020 instrument. Before measurements, 200 mg of sample were degassed in vacuum at 350°C for 4 h.

The morphology and structure of the catalyst surfaces was examined with a scanning electron microscope (FEI Quanta 200 FEG) using a backscattered electron detector.

For phase analysis and crystallite size calculations, X-ray powder diffraction (XRD) of fresh and used catalysts was performed using a Malvern PANanalytical MPD pro XRD (parameters: 45 kV anode, 40 mA (Cu-tube), 5°–100° 2Θ, 2.546° active length, 35 s per step, 25 min total duration). Diffractograms were refined with the Rietveld-method and the crystallite size was calculated according to the Scherrer equation using High Score Plus.

To evaluate the influence of the pyrolysis temperature on the surface characteristics of SiOC, hydrophobic and hydrophilic properties were determined by vapor adsorption using water and hexane. Therefore, SiOC powders pyrolyzed at 600 or 800°C, respectively, were dried at 100°C for 12 h. The sample mass was determined after drying and after keeping the sample in saturated H_2_O or hexane atmosphere for 24 h at room temperature.

The reducibility of the active sites was determined by temperature programmed reduction (H_2_-TPR) in hydrogen atmosphere, performed on a BELCATII instrument. For each measurement, 50–100 mg of sample were first pre-treated in Ar (30 min at 400°C, another 30 min at 50°C). Subsequently, the sample was heated to 1,000°C (heating ramp of 10°C/min) in a total gas flow of 50 mL/min (10 vol% H_2_ + 90 vol% Ar). The outlet gas was analysed using a TCD and the hydrogen consumption was determined.

To elucidate the formation and nature of adsorbed species during the methanation reaction, *in-situ* DRIFTS (Diffuse Reflectance Infrared Fourier Transform Spectroscopy) measurements were performed using a Bruker Vertex 70 infrared spectrometer. The device was equipped with a reaction cell from PIKE Technologies which is designed for temperature control and allows permanent gas flow through the catalyst bed. Each sample was pretreated in a total gas flow of 50 mL/min consisting of 10 vol% H_2_ and 90 vol% He at 400°C for 30 min. After cooling the sample to 30°C, a background spectrum was recorded in He. Then, the gas feed was switched to the reaction mixture (5 mL/min CO_2_, 20 mL/min H_2_ and 25 mL/min He) and the sample was heated to 400°C in steps of 50°C. After 30 min at each temperature step, spectra were recorded in the range of 900–4,000 cm^−1^ at a resolution of 4 cm^−1^.

### 2.4 Catalytic performance

CO_2_ methanation and stability tests were performed in a fixed bed steel reactor with an inner diameter of 6 mm at atmospheric pressure. 1 g of catalyst was mixed with 1 g of Al_2_O_3_ micro-spheres as an inert filler material. Prior to catalytic reaction, nickel sites were activated in H_2_ atmosphere (5 vol% H_2_ + 95 vol% He, 50 mL/min) at 400°C for 4 h. After pre-treatment, the gas flow (50 mL/min) was switched to the reaction feed consisting of CO_2_:H_2_:He = 1:4:5. The catalytic activity was evaluated at temperature steps of 50°C between 200°C and 400°C, with a dwell time of 2 h at each step. The outlet gas was analysed using an Inficon Fusion Micro GC equipped with a RT Molsieve 5A and a RT-Q bond module. CO_2_ conversion *X*_*CO2*_ and selectivity to methane *S*_*CH4*_ were calculated according to Equations 2 and 3) where [X] (X = CO_2_, CH_4_) represents the molar fraction of component X in the outlet gas.

$$X_{CO_{2}}\left(\%\right)=\frac{{\left[CO_{2}\right]}_{in}-{\left[CO_{2}\right]}_{out}}{{\left[CO_{2}\right]}_{in}}*100$$ (2)

$$S_{CH_{4}}\left(\%\right)=\frac{{\left[CH_{4}\right]}_{out}}{{\left[CO_{2}\right]}_{in}-{\left[CO_{2}\right]}_{out}}*100$$ (3)

## 3 Results and discussion

### 3.1 Surface properties

**FIGURE 1:**
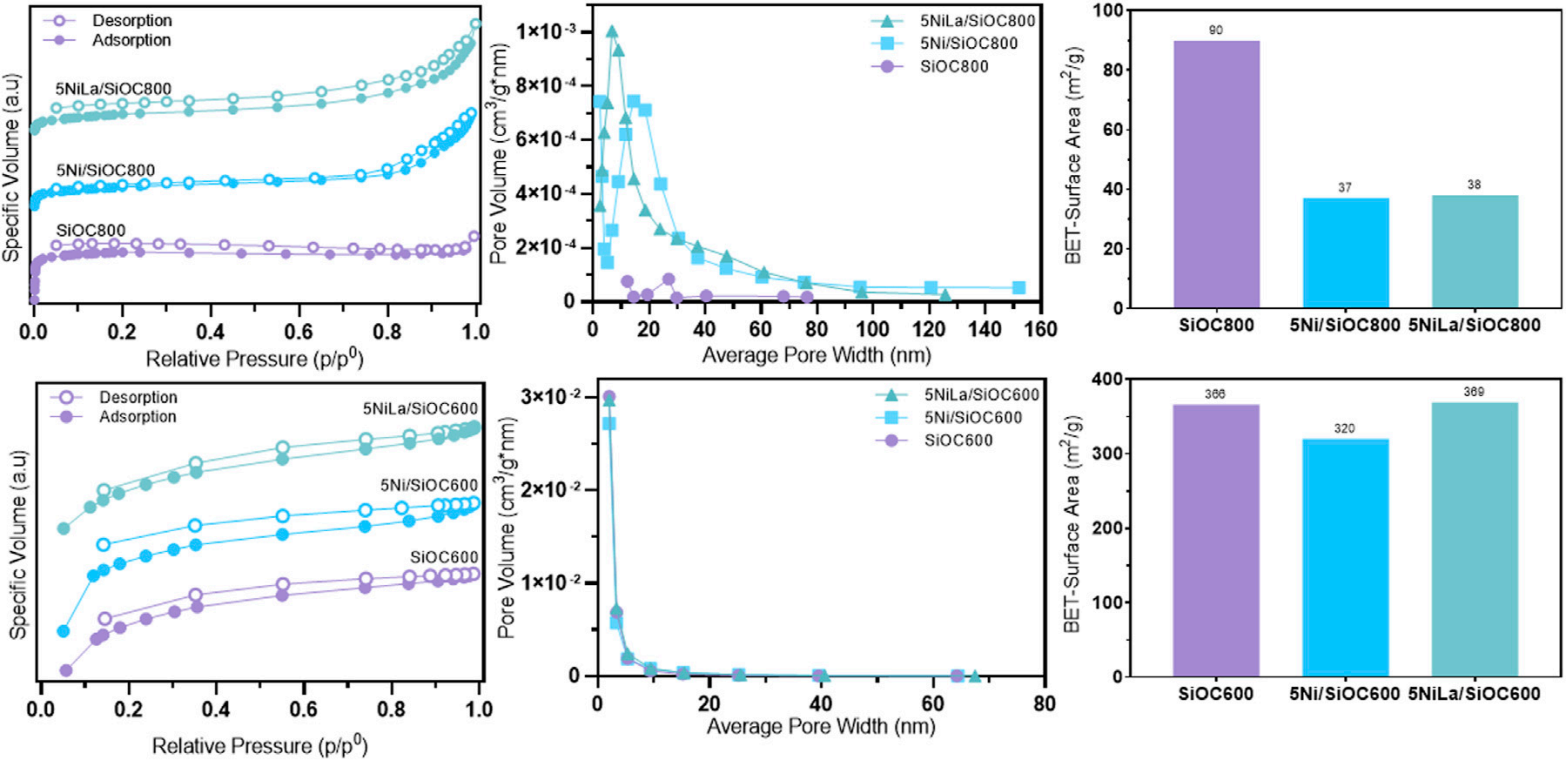
N_2_-adsorption-desorption isotherms, BJH pore size distribution and BET surface area of the support material and impregnated catalysts supported on SiOC pyrolyzed at 600°C (bottom row) and 800°C (top row).

N_2_ adsorption and desorption isotherms, pore size distribution, and BET surface area are presented in Figure 1. The BET values clearly show the influence of the pyrolysis temperature on the specific surface area: at lower pyrolysis temperatures, the surface area increases significantly. For catalytic applications, a high specific surface area combined with open, hierarchical porosity is desired to ameliorate both dispersion of active sites and adsorption capacities for reaction gases. When using polymer-derived SiOC as a catalyst carrier material, porosity is formed during the thermal treatment process due to gaseous decomposition of organic compounds as acrylates and/or methyl groups of Silres MK. At temperatures from 400°C to 800°C, volatile compounds such as CH_4_, CO, CO_2_, H_2_ and other hydrocarbons are released due to the cleavage of Si-H and C-H bonds (Macedo et al., 2019). At higher temperatures, starting from 800°C, transient porosity collapses due to viscous flow and diffusion. Also, evaporation-condensation reactions can take place leading to a consolidation of the material (Konegger et al., 2021). The specific surface area and porosity of polymer-derived SiOC pyrolyzed at 600 or 800°C, respectively, reflects the behaviour just described. At the lower pyrolysis temperature, SiOC exhibits a significantly higher surface area of 366 m^2^/g with an average pore size of 2.8 nm, while SiOC pyrolyzed at 800°C has a surface area of 90 m^2^/g and an average pore size of 21 nm. After impregnation, NiO- and La_2_O_3_-particles occupy pores of the substrate leading to a diminished surface area. The pore blocking seems to be more pronounced with the support material pyrolyzed at 800°C, as the larger pores within this material can be more easily penetrated by the NiO and La_2_O_3_ nanoparticles during impregnation.

**FIGURE 2:**
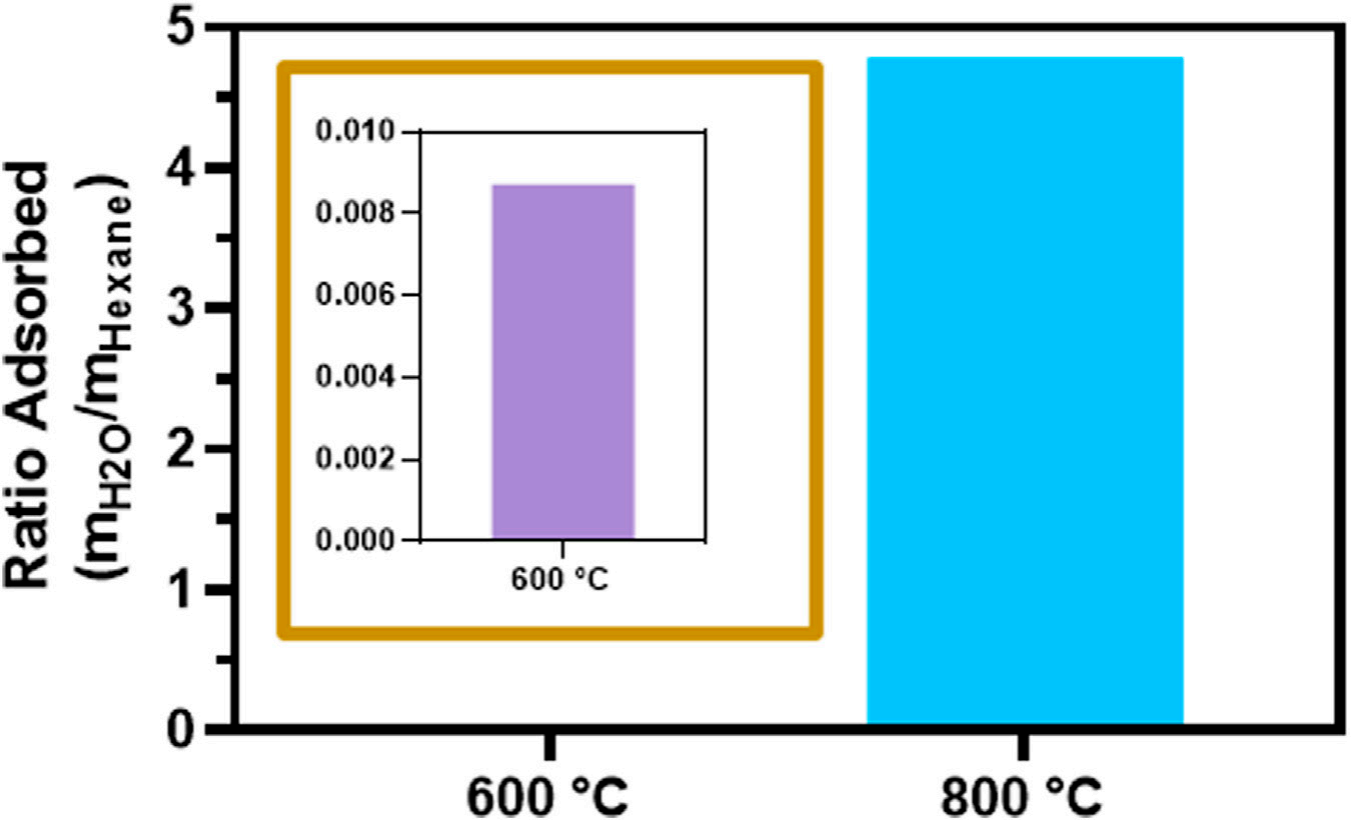
Water and hexane adsorption behaviour on SiOC pyrolyzed at different temperatures.

The results of water and hexane adsorption on SiOC supports pyrolyzed at 600°C and 800°C are depicted in Figure 2. The amount of water adsorbed on SiOC800 was orders of magnitude higher than for SiOC600, which highlights the influence of the pyrolysis temperature on the material properties. At lower temperatures, more organic groups remain on the surface of SiOC, creating a hydrophobic material. With increasing temperatures, these organic groups are decomposed and the material properties becomes more hydrophilic (Macedo et al., 2019; Schumacher et al., 2020). With respect to an application as catalyst support material for CO_2_ methanation, a hydrophobic surface can be advantageous as water is a by-product in this reaction. If the resulting water can be kept away from the catalysts surface, more adsorption capacity can be provided for the reaction gases, and the catalytic activity can be enhanced.

**FIGURE 3:**
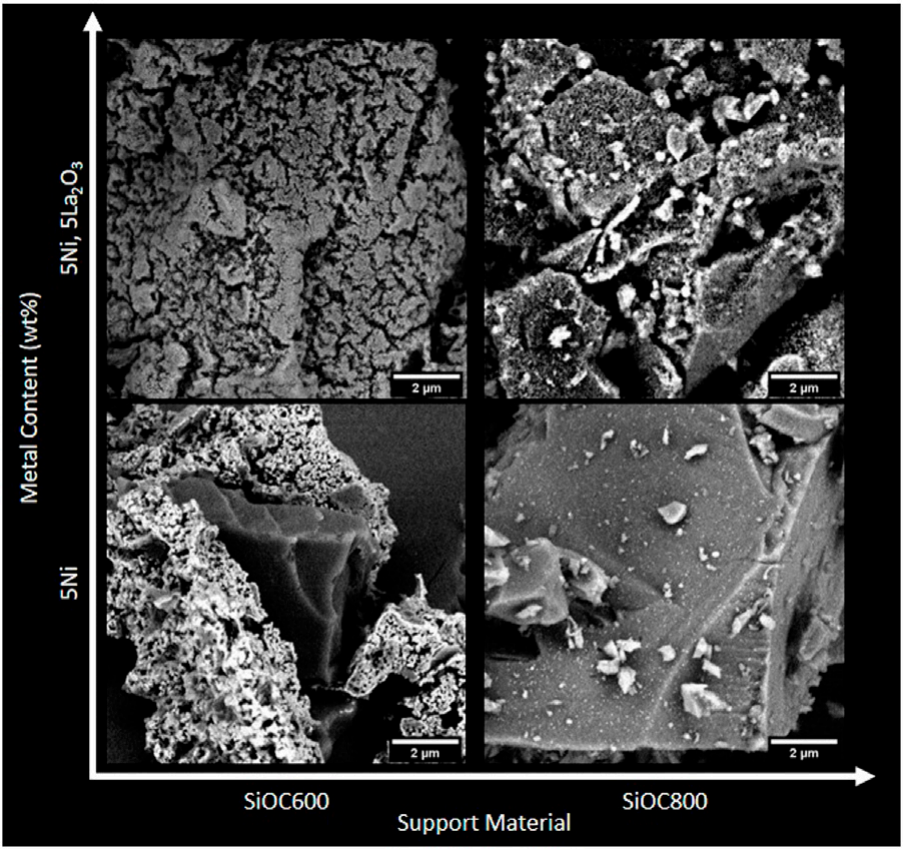
SEM images (BSE-contrast) of catalysts with (top row) and without (bottom row) La-modification as a function of pyrolysis temperature of the support material.

An overview of SEM images of impregnated catalysts with different Ni loadings on the various support materials is presented in Figure 3. In catalysts containing 5 wt% Ni, the surface is covered by feather-like structures. By means of backscattered electron (BSE) contrast, these structures can be attributed to Ni. At first sight, it appears that Ni sites on La-modified supports are finely dispersed. The surface of modified samples seems to be more even without the fine feather-like structures which can be observed for the unmodified catalysts. La-modified catalysts are prepared by a double impregnation process where the support is impregnated with La(NO_3_)_3_ first. After calcination, the modified support material is impregnated again with Ni(NO_3_)_2_. Combined with the hydrophobic properties of SiOC600, this procedure could lead to the coral-like features which can be observed on the surface of 5NiLa/SiOC600. Another result of the two-step preparation process is a slightly increased surface area in modified samples.

### 3.2 Reducibility

**FIGURE 4:**
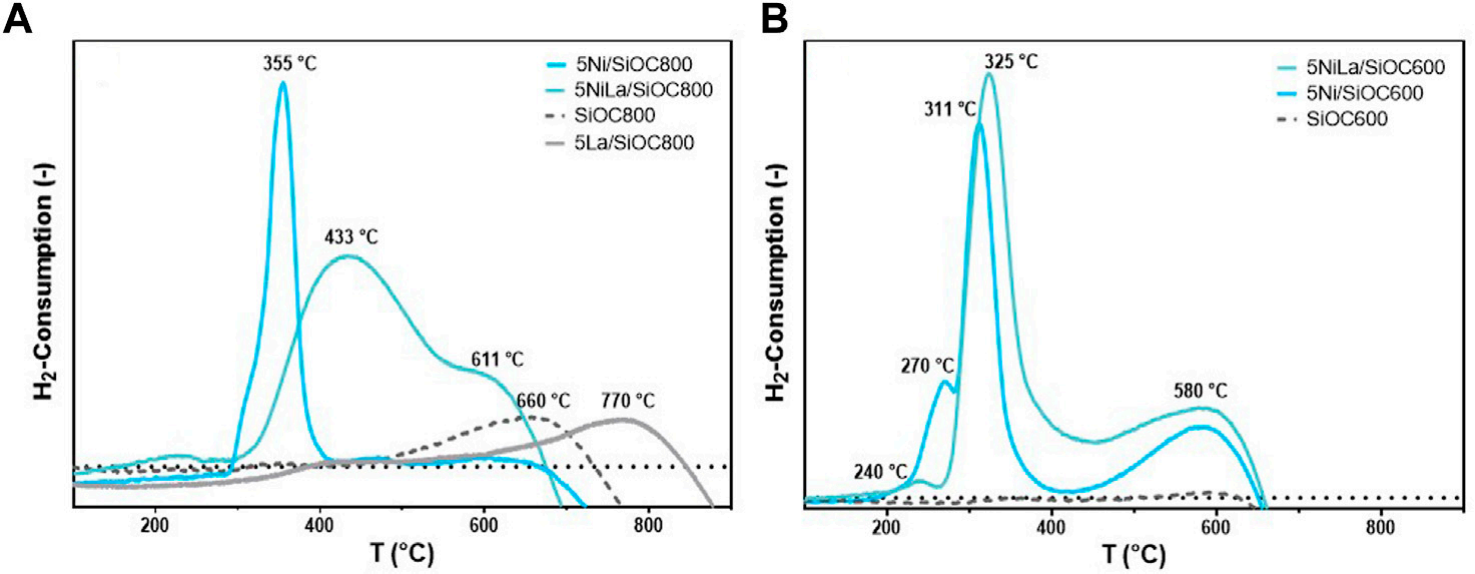
H_2_-TPR profiles of catalysts with and without La-modification supported on SiOC pyrolyzed at 800°C **(A)** and 600°C **(B)**.

Figure 4 shows the TPR profiles of catalysts supported on SiOC600 and SiOC800 with and without La modification. H_2_-TPR measurements provide information about the reducibility of the active phase but also on the strength of interactions between metal oxides and the support material. It is generally accepted that, since the reduction of NiO to Ni is a single step process (NiO + H_2_ → Ni^0^+H_2_O), irregular shapes of hydrogen consumption peaks can be attributed to differences in the NiO-support interactions (Branco et al., 2020; Mihet et al., 2021). The unmodified catalyst supported on polymer-derived SiOC pyrolyzed at 800°C (Figure 4A) shows a single H_2_ consumption peak at 355°C. With introduction of La_2_O_3_ onto the support material, the signal broadens, and the peak shifts to a higher temperature of 433°C, with a pronounced shoulder at 611°C. These phenomena indicate an increased interaction of the La_2_O_3_-SiOC800 support with NiO. The reference material (SiOC800) exhibits a peak at 660°C, most likely stemming from degradation of the support material at higher temperatures. After support modification with La_2_O_3_, this peak shifts to 770°C, which indicates an increased stability of the support material. Since SiOC pyrolyzed at 600°C shows no activity in the H_2_-TPR itself, the peaks at 270, 311°C and 580°C in the sample containing 5 wt% Ni can be attributed to NiO particles with different reducibility. The additional peak at 580°C indicates the presence of a NiO species in stronger interaction with the support material, which leads to an increased stability against sintering of the active phase in this sample (Mihet et al., 2021). Crystallite size determinations of the fresh and used catalyst in Table 1 confirm this observation as no significant crystallite growth occurred during catalytic reaction. The La-modified sample shows a similar behaviour. Here, the main hydrogen consumption takes place between 325°C and 580°C, while the peak intensity at 270°C is significantly lower. This indicates a higher proportion of harder reducible NiO particles due to higher interaction with the support material.

**TABLE 1:** Ni crystallite size estimated using the Scherrer equation of fresh and used (after catalytic tests and after stability tests) catalysts with and without La-modification.

Support material	Ni content (wt%)	La_2_O_3_ content (wt%)	Crystallite size (nm)		
			Fresh	After catalytic reaction (2 h at T_max_)	After stability test (50 h at T_max_)
SiOC800	5	0	11.83 ± 0.09	39.42 ± 0.48	56.65 ± 0.32
		5	5.21 ± 0.005	8.09 ± 0.19	7.22 ± 0.00
SiOC600	5	0	34.20 ± 0.4	36.26 ± 0.21	28.93 ± 0.10
		5	9.44 ± 0.14	10.87 ± 0.09	9.01 ± 0.08

### 3.3 Phase analysis and crystallite size

**FIGURE 5:**
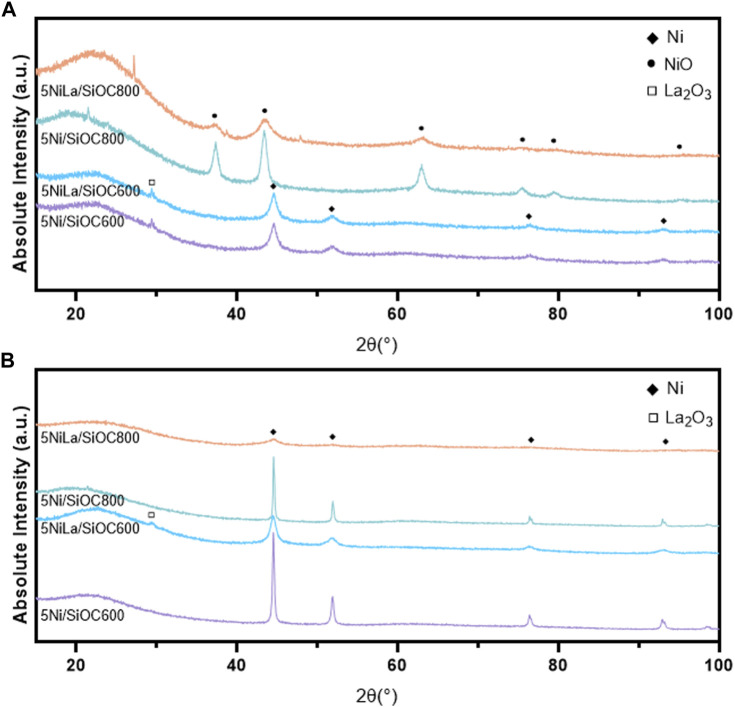
X-ray powder diffraction patterns of catalysts before **(A)** and after **(B)** catalytic test reaction.

Figure 5 shows the powder diffraction patterns of fresh and used catalysts. Powder diffraction patterns of unimpregnated support materials can be found in Supplementary Figures S2, S3. Fresh samples supported on SiOC800 contain NiO (JCPDS 04-013-0890) as sole Ni-containing phase, which is fully converted to metallic Ni (JCPDS 00-004-0850) after pretreatment procedure in H_2_ and reaction. In contrast to SiOC800, catalysts supported on SiOC600 contain only metallic nickel. This can be explained by different calcination conditions: While SiOC800-supported samples were calcined in air, catalysts based on SiOC600 were calcined in Ar atmosphere where the formation of NiO was suppressed (Mihet et al., 2021). While the Ni phases can be clearly identified, most of the diffraction patterns do not show a La_2_O_3_ reflex as this phase may be highly dispersed. The existence of La in these samples could be confirmed by EDX measurements, for the sake of brevity, their results are reported in Supplementary Figures S4, S5 and Supplementary Tables S1, S2.

Previous studies reported that the introduction of La_2_O_3_ to Ni catalysts on several support materials such as SiC or SiO_2_ led to a higher dispersion of the active phase and thus enhances both stability and performance during catalytic reaction (Zhi et al., 2011; Mihet et al., 2021). These results could also be confirmed in this work: Compared to unmodified catalysts, XRD measurements reveal a drastically smaller crystallite size for Ni on La-modified supports, indicating a high dispersion of this phase. The Ni crystallite size of fresh and used catalysts with and without La-modification is listed in Table 1. When comparing the fresh catalysts, it is noticeable that the active phase on the La_2_O_3_ modified support exhibits significantly smaller crystallites than in the unmodified sample. While the crystallite size of the used unmodified catalyst supported on SiOC800 is 3.3 times higher than in the fresh sample, the crystallite growth during the reaction could be reduced to 55% when using a La-modified support. Regardless of the modification, no significant crystallite growth can be observed in the used catalyst impregnated on SiOC600. Here, it must be noted that the fresh catalyst without La-modification showed significantly larger Ni-crystallites than the fresh sample on SiOC800. It can be assumed that sintering of the Ni-particles already occurred during calcination.

### 3.4 *In-situ* DRIFTS studies of SiOC800 supported samples

**FIGURE 6:**
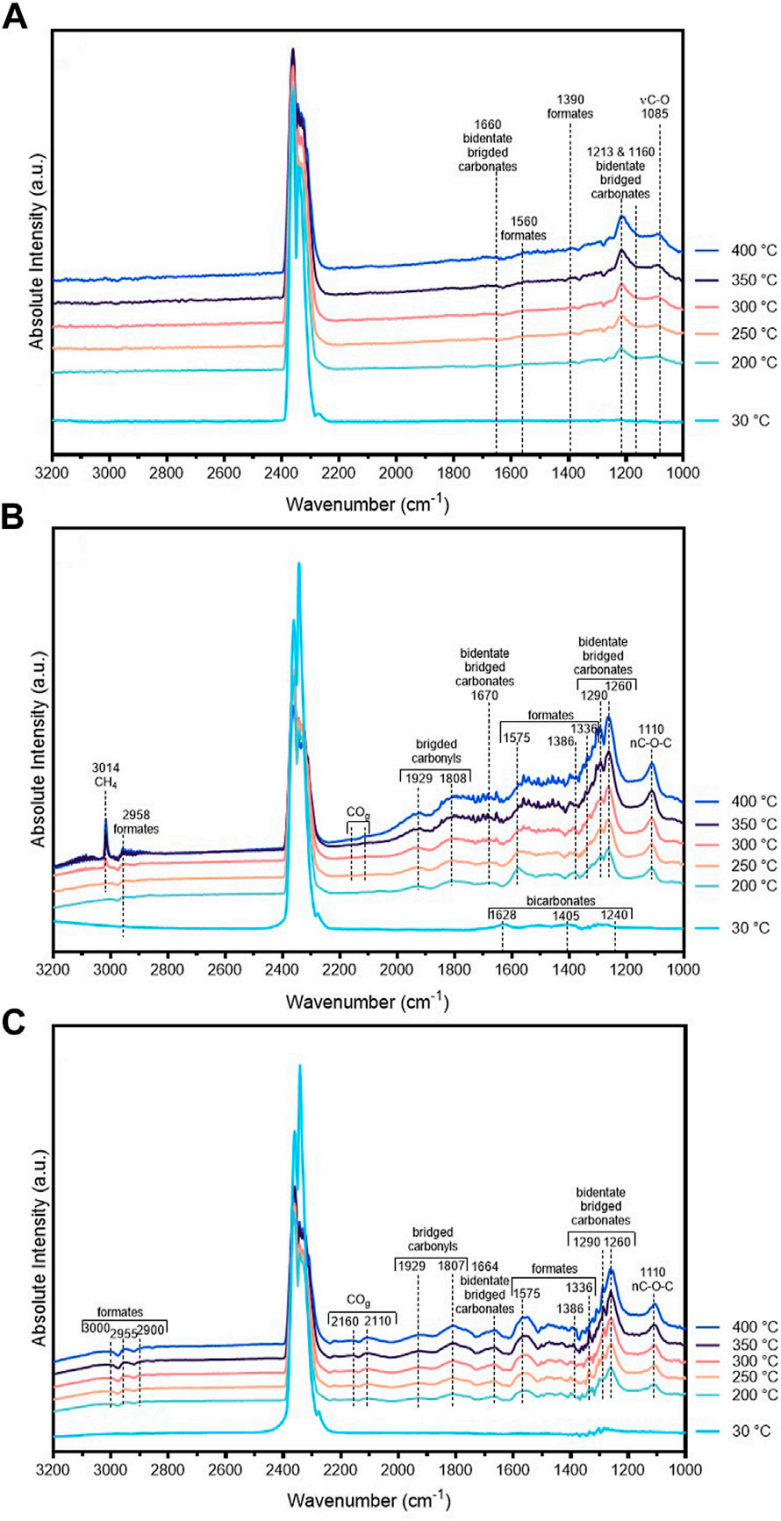
*In-situ* DRIFTS experiments of SiOC800-supported samples under reaction conditions (He/H_2_/CO_2_ = 5:4:1) at different temperatures: 5Ni/SiOC800 **(A)**, 5NiLa/SiOC800 **(B)** and 5La/SiOC800 **(C)**. For each measurement, a background spectrum recorded under He at room temperature was subtracted.

In order to elucidate the nature of adsorbed species on SiOC800-supported catalysts under reaction conditions at different temperatures, *in-situ* DRIFTS studies were carried out with samples containing 5 wt% Ni as well as with La-modified samples with and without Ni, the resulting spectra being compiled in Figure 6. Identification of adsorbed species was carried out based on wavenumber ranges published in literature (Cárdenas-Arenas et al., 2020). Due to the presence of CO_2_ in the reaction gas mixture, an intense double band occurs at 2,350 cm^−1^. Starting at 200°C, the band occurring at 1,085 cm^−1^ in the unmodified sample (Figure 6A) can be attributed to νC-O vibrations, while the bands at 1,560 and 1,390 cm^−1^ can be assigned to formates adsorbed onto Ni^0^ (Westermann et al., 2015; Cárdenas-Arenas et al., 2020). A broad signal at 1,213 cm^−1^ gaining intensity with increasing temperature and the signal at 1,660 cm^−1^ can likely be assigned to bidentate carbonates in the bridged configuration (Föttinger et al., 2008; Haghofer et al., 2012; Föttinger et al., 2017; Cárdenas-Arenas et al., 2020).

In the La-modified Ni sample (Figure 6B), a band occurs at 3,014 cm^−1^ from 250°C on and gains intensity at higher temperatures. This can be related to the formation of methane in the gas phase. Besides the CH_4_ signal, also small rotational-vibrational signals occur which refers to the existence of water in the gas phase, another indication for the onset point of the methanation reaction. At 30°C, bands can be observed at 1,240, 1,405 and 1,628 cm^−1^ which can be assigned to bicarbonate species. These bands disappear at higher temperatures. From 200°C on, the CH–stretching of formates can be observed at 2,958 cm^−1^. Also, the signals at 1,575, 1,386 and 1,336 cm^−1^ can be related to this species, here, the asymmetric νCOO and symmetric stretching modes and the δCH are visible. The band at 1,386 cm^−1^ corresponds to the symmetric νCOO vibration of formates. In this sample, also bidentate carbonates in bridging configuration (1,670 cm^−1^ and 1,260–1,290 cm^−1^), νC-O-C (1,110 cm^−1^), and bridged carbonyls (1808 & 1929 cm^−1^) can be observed (Föttinger et al., 2008; Haghofer et al., 2012; Föttinger et al., 2017; Cárdenas-Arenas et al., 2020).

To investigate the role of La_2_O_3_ in this reaction, a spectrum of the La-modified support material without nickel was recorded (Figure 6C). From a temperature of 200°C on, this sample also shows formate species in the range of 2,900–3,000 cm^−1^. Also, the corresponding signals at 1,575, 1,386 and 1,336 cm^−1^ are visible. In contrast to the sample containing also Ni, the formation of gaseous carbon monoxide can be observed at 2,110 and 2,160 cm^−1^ in this sample. Like the modified catalyst in Figure 6B, this sample also shows bidentate bridged carbonates (1,260, 1,290 and 1,664 cm^−1^) and bridged carbonyls (1807 and 1929 cm^−1^).

**FIGURE 7:**
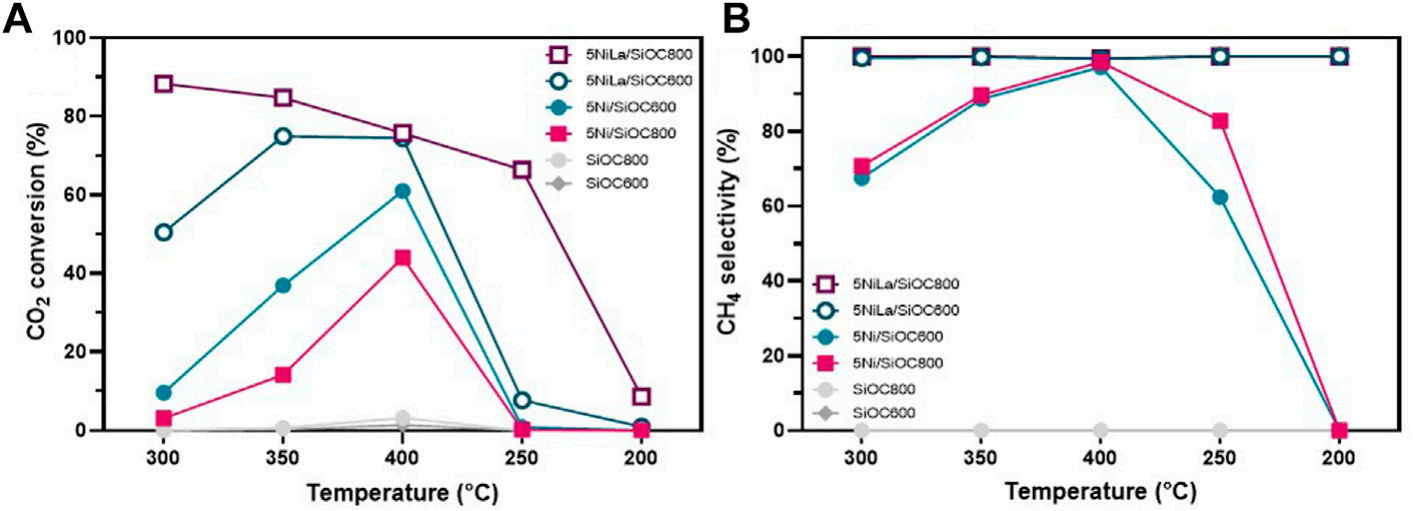
CO_2_ conversion **(A)** and CH_4_ selectivity **(B)** of samples on SiOC pyrolyzed at different temperatures, with and without La-modification. Please note that the *x*-axis is labelled according to the order of the temperature steps performed.

Catalytic test reaction (Figure 7) showed an onset point for the methanation reaction at 350°C for the unmodified SiOC800-supported catalyst while with La-modification, this onset temperature shifts to 250°C. The *in-situ* DRIFTS spectrum of 5NiLa/SiOC800 (Figure 6B) features two bands at 1,405 and 1,628 cm^−1^ at room temperature, which can be assigned to bicarbonate species. This observation supports the suggestion that with additional La_2_O_3_ modification, the adsorption of CO_2_ is favoured.

At higher temperatures, more adsorbed carbon species occurred such as bidentate carbonates (1,660, 1,260 & 1,290 cm^−1^). At 1808 and 1929 cm^−1^, bridged carbonyls can be observed which indicate CO also adsorbed onto Ni sites. At a temperature of 200°C, bands at 1,575, 1,386 and 1,336 cm^−1^ occur which can be attributed to formate species. These bands become weaker with increasing temperature while simultaneously, a band at 3,014 cm^−1^ arises, indicating the formation of gaseous CH_4_. As it is generally accepted that formates are intermediate species during the CO_2_ methanation reaction, this observation suggests that CO_2_ is already activated at lower temperatures (Haghofer et al., 2012; Aldana et al., 2013; Cárdenas-Arenas et al., 2020; Huang et al., 2021). On the other hand, the spectrum of 5La/SiOC800 (Figure 6C) shows that formates are generally created once CO_2_ is chemisorbed. These formates can either hydrogenate and form CH_4_ as it is the case for 5NiLa/SiOC800, or they can hydrogenate and dissociate into CO and H_2_O as it can be observed for 5La/SiOC800 where a double band at 2,110 and 2,160 cm^−1^ indicates the formation of gaseous CO. This effect is also evidenced by catalytic testing reactions, where the same sample exhibited a CO_2_ conversion up to 1.2%, but only carbon monoxide was produced. The unmodified SiOC800-supported catalyst in Figure 6A showed less adsorbed species and neither CH_4_ nor CO formation could be observed which suggests that the catalytic activity here was below the detection threshold. In this sample, the formation of formates (1,560 and 1,390 cm^−1^), and bidentate bridged carbonates (1,660 and 1,213 cm^−1^) could be observed starting from 200°C.

### 3.5 Catalytic performance

Figure 7 presents a comparison of the SiOC-supported catalysts with different pyrolysis temperatures of the support material. During the catalytic test reaction, metallic nickel is present in all samples due to pretreatment procedure in H_2_ prior to catalytic reaction. It can be observed that the catalytic activity was increased in the sample supported on SiOC600. Compared to the SiOC800-supported sample, the CO_2_ conversion of the catalyst on SiOC600 is 2.6 and 1.4 times higher at reaction temperatures of 350°C and 400°C, respectively. The selectivity to methane, which is shown in Figure 7B, is comparable for both catalysts. At a reaction temperature of 400°C, the blank support material pyrolyzed at 800°C shows a CO_2_ conversion of 3.2%, while the reference pyrolyzed at 600°C reaches a value of 1.4% whereby only carbon monoxide was produced. The hydrophobic properties of SiOC pyrolyzed at 600°C offer an advantage for the CO_2_ methanation as water is a by-product of this reaction. If the resulting water can be kept away from the catalysts surface, more adsorption capacity can be provided for the reaction gases and the catalytic activity can be ameliorated.

With additional La_2_O_3_ modification, the catalyst supported on SiOC pyrolyzed at 800°C exhibited an outstanding catalytic performance in the temperature range of 250°C–350°C. Here, a CO_2_ conversion of 88% could be achieved at a reaction temperature of 300°C, while the activity of the unmodified catalyst on the same support is negligible at this point. Although the unmodified sample supported on SiOC600 shows a higher CO_2_ conversion than the SiOC800 supported catalyst, which could be further increased by La_2_O_3_ modification, the performance of the La-modified catalyst on SiOC800 could not be reproduced here. The remarkable activity of La-modified catalysts is due to their structural characteristics such as an increased specific surface area, low crystallite size, high stability of the Ni phase and a high dispersion of metal centres. Thus, a high number of active sites for hydrogen adsorption is provided.

The generally accepted mechanism of CO_2_ methanation is that CO_2_ is adsorbed onto the catalyst surface and dissociates to CO. Subsequently, it gets hydrogenated in several intermediate steps to CH_4_ (Zhi et al., 2011; Aldana et al., 2013; Aziz et al., 2014; Huang et al., 2021). Here, La_2_O_3_ plays an important role which is described as follows by *Zhi et al* (Zhi et al., 2011): On the one hand, La_2_O_3_ provides additional adsorption sites. As it is a basic material, the adsorption and dissociation of CO_2_ is favoured. On the other hand, La_2_O_3_ contributes d-electron density to Ni sites and thus adsorbed CO_2_ can be activated by means of d-electron donation to the antibonding π* orbit of CO_2_. In consequence, the Ni–C bond becomes stronger while the C–O bond is weakened, which facilitates hydrogenation. These explanations are reflected in the enhanced catalytic activity of lanthanum promoted catalysts.

**FIGURE 8:**
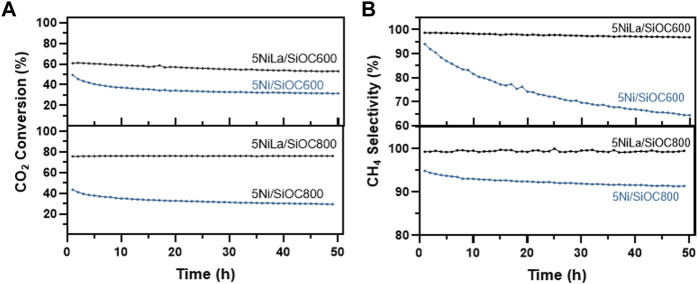
CO_2_ conversion **(A)** and CH_4_ selectivity **(B)** of catalysts with and without La-modification over the course of 50 h at 400°C.

To evaluate their stability, catalysts (5 wt% Ni, modified/unmodified with La) were tested under harsh conditions at a reaction temperature of 400°C reaction for 50 h. The results are compiled in Figure 8. For 5Ni/SiOC800, the CO_2_ conversion decreases rapidly from 43% to 35% within the first 10 h. After 50 h, this sample showed only 29% CO_2_ conversion, which corresponds to a decrease in activity of one-third. In case of the catalyst supported on unmodified SiOC pyrolyzed at 600°C, the activity decreases significantly within 50 h from 49.3% to 31.5%. While the catalytic activity of 5NiLa/SiOC800 remained stable throughout the temperature profile, the La-modified SiOC600-supported sample showed a slow decrease in activity from a CO_2_ conversion of 60.6% in the beginning to 53% after 50 h. The support modification promoted not only the catalytic activity in the test reaction but also the catalyst stability at harsh conditions. After 50 h at 400°C, unmodified catalysts showed only two-thirds of their initial activity. While the unmodified SiOC600 supported catalyst showed an enhanced activity during the catalytic test reaction due to remaining organic groups on the support surface resulting in hydrophobic properties, these compounds were decomposed successively at high temperature leading to a decrease in activity.

## 4 Conclusion

Depending on the pyrolysis temperature of the support material, Ni-SiOC catalysts showed catalytic activity in the Sabatier reaction with CO_2_ conversions of 44% (5Ni/SiOC800) and 61% (5Ni/SiOC600) and selectivity to methane of 98.5% (5Ni/SiOC800) and 97% (5Ni/SiOC600) at 400°C reaction temperature. A lower pyrolysis temperature of the support material of 600°C offered advantages for catalytic applications such as a high surface area, enhanced stability of active sites and a hydrophobic surface, at least in short term testing conditions. With La_2_O_3_ modification of the support material, both additional surface area and adsorption capacity for CO_2_ could be provided. In all the modified catalysts, crystallite size of the active phase was significantly lower and remained stable not only during catalytic test reactions but also after exposure to 400°C for 50 h. An outstanding catalytic performance could be achieved by support modification of SiOC800: This catalyst showed activity from 200°C on and exhibited both highest CO_2_ conversion (66%–88%) and CH_4_ selectivity (>99%) amongst all catalysts between reaction temperatures of 250°C–350°C. This work demonstrates the highly promising nature of polymer-derived SiOC as support material for heterogeneous catalysis, in particular owing to the high prospective flexibility in shaping provided by the photocurability of the starting material used.

## Data Availability

The original contributions presented in the study are included in the article/Supplementary Material, further inquiries can be directed to the corresponding author.
