# Supplementary material for: Polymer-derived SiOC as support material for Ni-based catalysts: CO2 methanation performance and effect of support modification with La2O3
Source: Front Chem. 2023 Mar 22;11:1163503. doi: 10.3389/fchem.2023.1163503 (PMC10073567; doi:10.3389/fchem.2023.1163503)
Supplement: Supplementary file 1 [file DataSheet1.docx]

Polymer-derived SiOC as support material for Ni-based catalysts: CO_2_ methanation performance and effect of support modification with La_2_O_3_

Supporting Information

E. Szoldatits^1^, J. Essmeister^2^, L. Schachtner^2^, T. Konegger^2^, K. Föttinger^1^*

^1^Institute of Materials Chemistry, Getreidemarkt 9, 1060 Vienna, TU Wien, Austria

^2^Institute of Chemical Technologies and Analytics, Getreidemarkt 9, 1060 Vienna, TU Wien, Austria

**Experimental Section**


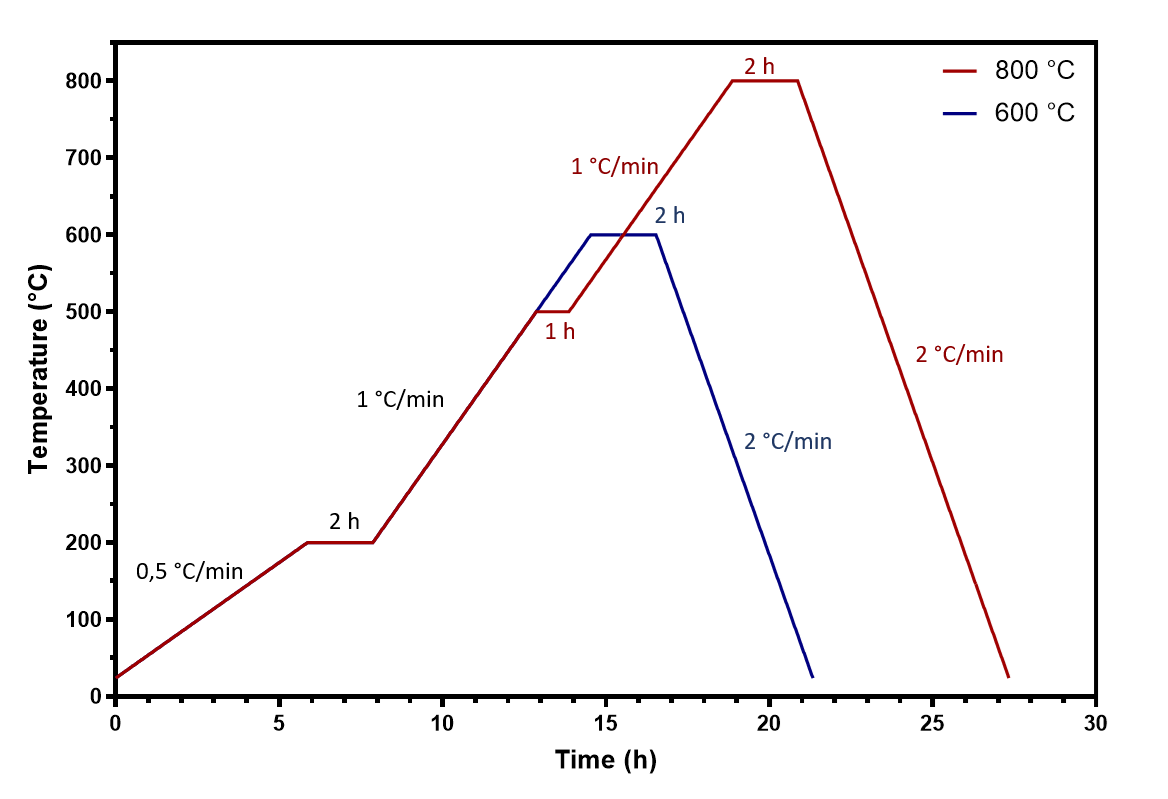


Figure S1: Temperature profile of pyrolysis to convert preceramic polymer into SiOC as support material


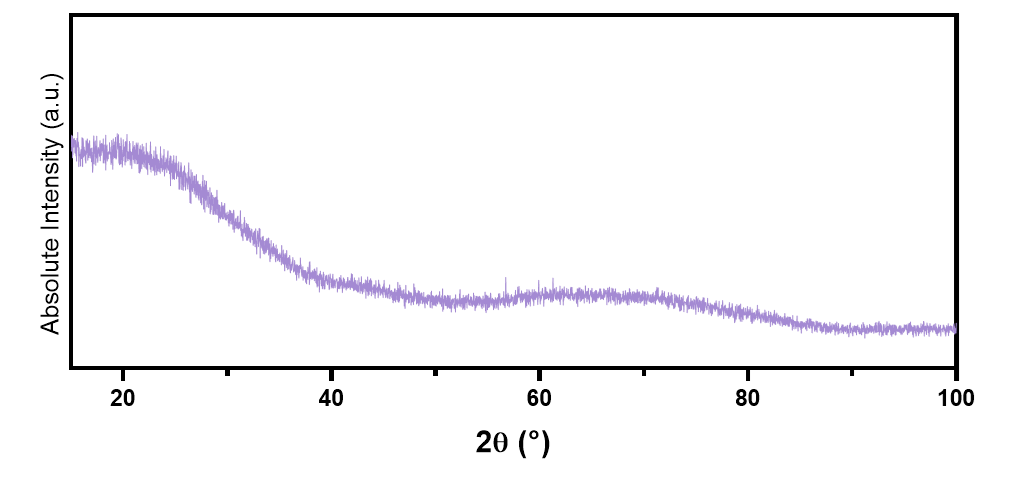
**XRD measurements of SiOC support**

Figure S2: Powder diffraction pattern of SiOC support pyrolyzed at 600 °C


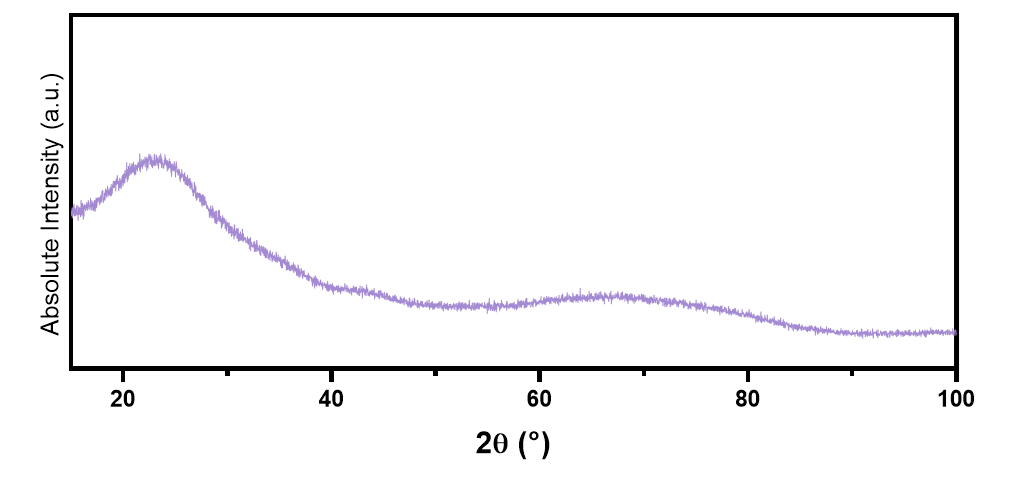


Figure S3: Powder diffraction pattern of SiOC support pyrolyzed at 800 °C

**EDX measurements**

5NiLa/SiOC600Table S1: Results of EDX measurement of 5NiLa/SiOC600 contained in Figure S2

Figure S4: EDX measurement of 5NiLa/SiOC600


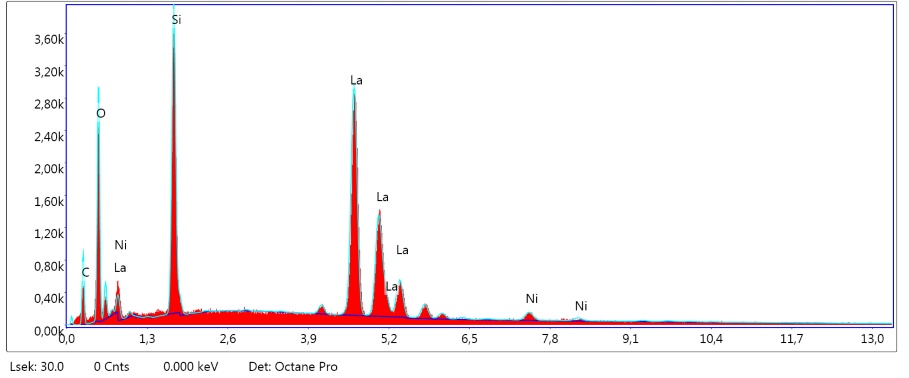

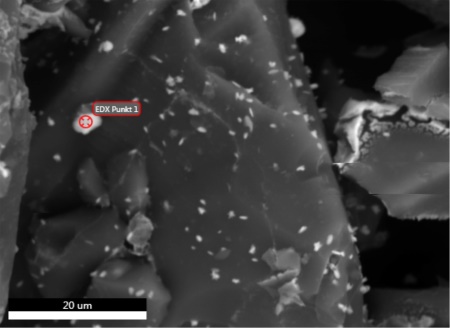


| Element | weight% | Atom% | Net. Int. |
| --- | --- | --- | --- |
| C K | 11.21 | 31.94 | 117.04 |
| O K | 16.64 | 35.61 | 483.94 |
| SiK | 14.38 | 17.53 | 987.97 |
| LaL | 55.75 | 13.74 | 1189.23 |
| NiK | 2.02 | 1.18 | 49.93 |

5NiLa/SiOC800Table S2: Results of EDX measurement of 5NiLa/SiOC800 contained in Figure S3

Figure S5: EDX measurement of 5NiLa/SiOC800


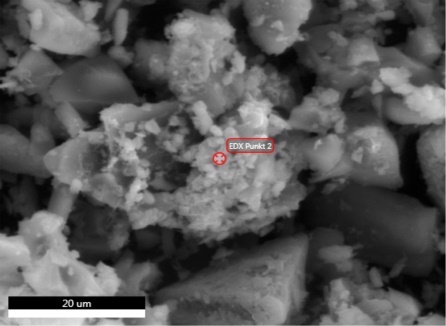

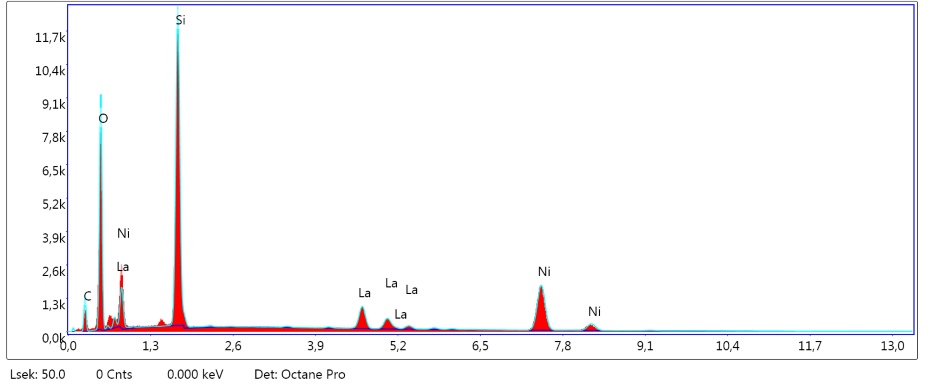


| Element | weight% | Atom% | Net. Int. |
| --- | --- | --- | --- |
| C K | 15.05 | 27.86 | 112.75 |
| O K | 33.14 | 46.06 | 931.22 |
| SiK | 20.87 | 16.52 | 1909.92 |
| LaL | 9.92 | 1.59 | 217.34 |
| NiK | 21.03 | 7.97 | 542.40 |
